# Design of a New [*PSI*^+^]-No-More Mutation in *SUP35* With Strong Inhibitory Effect on the [*PSI*^+^] Prion Propagation

**DOI:** 10.3389/fnmol.2019.00274

**Published:** 2019-11-19

**Authors:** Lavrentii G. Danilov, Andrew G. Matveenko, Varvara E. Ryzhkova, Mikhail V. Belousov, Olga I. Poleshchuk, Daria V. Likholetova, Petr A. Sokolov, Nina A. Kasyanenko, Andrey V. Kajava, Galina A. Zhouravleva, Stanislav A. Bondarev

**Affiliations:** ^1^Department of Genetics and Biotechnology, St. Petersburg State University, St. Petersburg, Russia; ^2^Laboratory for Proteomics of Supra-Organismal Systems, All-Russia Research Institute for Agricultural Microbiology (ARRIAM), St. Petersburg, Russia; ^3^Department of Molecular Biophysics and Polymer Physics, St. Petersburg State University, St. Petersburg, Russia; ^4^Centre de Recherche en Biologie cellulaire de Montpellier (CRBM), UMR 5237 CNRS, Université Montpellier, Montpellier, France; ^5^Institut de Biologie Computationnelle (IBC), Universitè Montpellier, Montpellier, France; ^6^Laboratory of Amyloid Biology, St. Petersburg State University, St. Petersburg, Russia

**Keywords:** [*PSI*^+^], amyloid, ArchCandy, prion, *Saccharomyces cerevisiae*, *SUP35* mutation, superpleated-β-structure

## Abstract

A number of [*PSI*^+^]-no-more (PNM) mutations, eliminating [*PSI*^+^] prion, were previously described in *SUP35*. In this study, we designed and analyzed a new PNM mutation based on the parallel in-register β-structure of Sup35 prion fibrils suggested by the known experimental data. In such an arrangement, substitution of non-charged residues by charged ones may destabilize the fibril structure. We introduced Q33K/A34K amino acid substitutions into the Sup35 protein, corresponding allele was called *sup35-M0*. The mutagenized residues were chosen based on ArchCandy *in silico* prediction of high inhibitory effect on the amyloidogenic potential of Sup35. The experiments confirmed that Sup35-M0 leads to the elimination of [*PSI*^+^] with high efficiency. Our data suggested that the elimination of the [*PSI*^+^] prion is associated with the decreased aggregation properties of the protein. The new mutation can induce the prion with very low efficiency and is able to propagate only weak [*PSI*^+^] prion variants. We also showed that Sup35-M0 protein co-aggregates with the wild-type Sup35 *in vivo*. Moreover, our data confirmed the utility of the strategy of substitution of non-charged residues by charged ones to design new mutations to inhibit a prion formation.

## 1. Introduction

Prions are self-propagating and transmissible protein isoforms that cause fatal neurodegenerative disease in humans or heritable traits in lower eukaryotes. The most known hallmark of almost all prions is a formation of amyloid aggregates (Liebman and Chernoff, 2012). These aggregates have a set of specific properties, such as resistance to detergents and proteases, interaction with dyes Thioflavin T and S, birefringence when stained with the Congo Red dye, and cross-β-structure (Baxa et al., 2006). The first discovered prion, PrP^Sc^ (“prion protein” scrapie), causes severe infectious neurodegenerative diseases in mammals (Prusiner, 2013). Discovery of prions in lower eukaryotes (Wickner, 1994) revealed that this phenomenon is widespread and based on common mechanisms. Thus, unicellular organisms such as yeast can be used for investigation of prionization and the data obtained can be extrapolated to mammalian prions (Liebman and Chernoff, 2012).

One of the best-studied prions to date is [*PSI*^+^], an isoform of Sup35 protein (Cox, 1965; Wickner, 1994; Wickner et al., 1995), which is a eukaryotic release factor 3 (Stansfield et al., 1995; Zhouravleva et al., 1995). This protein is divided into three domains (N, M, and C) (Kushnirov et al., 1988). The C-terminal part of Sup35 contains four GTP binding sites (Stansfield et al., 1995; Zhouravleva et al., 1995) and it is essential for the cell viability and termination of translation (Ter-Avanesyan et al., 1993). N-domain is required for [*PSI*^+^] maintenance (Ter-Avanesyan et al., 1994) and formation of stress-inducible condensates (Franzmann et al., 2018). N-domain consists of two parts — the Q/N rich segment (1–39 aa) and the oligopeptide repeats region (OR), containing one incomplete and five complete repeats (40–112 aa) (Kushnirov et al., 1988). The charged M-domain represents an unfolded linker that also affects [*PSI*^+^] prion maintenance (Liu et al., 2002; Helsen and Glover, 2012).

Cells bearing [*PSI*^+^] prion have a reduced amount of monomeric Sup35 protein, that increases the frequency of the read-through of premature stop codons (Liebman and Chernoff, 2012). Nonsense mutation *ade1-14*, which lead to the synthesis of a truncated non-functional Ade1 protein and to the inability of cells to synthesize adenine, is often used to test for the nonsense suppression caused by the prion. The accumulation of the adenine biosynthesis intermediate results in the red color of colonies growing on the 1/4 YEPD medium. The appearance of [*PSI*^+^] prion leads to the suppression of *ade1-14* nonsense mutation and the formation of full-length Ade1. Phenotypically it can be detected by growth on media lacking adenine and the white colony color. This manifestation can vary depending on the structure of Sup35 aggregates (prion variant), templated upon prion propagation. The term “variant” is used hereafter for different prion variants, and “strain” — only for yeast strains. Cells bearing weak variants of the [*PSI*^+^] prion demonstrate weak growth on adenineless media, *i.e*., weak nonsense suppression, while the strong [*PSI*^+^] variants lead to almost complete masking of the *ade1-14* mutant phenotype (Liebman and Chernoff, 2012).

Different approaches may be used for investigation of Sup35 aggregates in [*PSI*^+^] cells. They can be decorated by transiently overproduced Sup35NM-GFP and visualized with fluorescence microscopy (Osherovich et al., 2004). Sup35 aggregates can also be directly analyzed with biochemical approaches: differential centrifugation, SDD-AGE (Kryndushkin et al., 2003) or modifications of SDS-PAGE (Kushnirov et al., 2006).

Oligopeptide repeats in the Sup35 N-domain significantly affect [*PSI*^+^] prion maintenance. At least two first ORs are essential for the prion propagation (Liu and Lindquist, 1999; Osherovich et al., 2004; Shkundina et al., 2006). At the same time OR expansion leads to increased fragmentation of Sup35 aggregates, while a decrease in the number of repeats has an opposite effect (Langlois et al., 2016). Previously, using the T-REKS algorithm (Jorda and Kajava, 2009), we identified an additional OR in the Sup35 N-domain, located from 28 to 40 amino acid residues (Bondarev et al., 2013). The mutation within this OR, named *sup35-M0*, was designed based on the model of a superpleated-β-structure, proposed for Sup35 aggregates (Kajava et al., 2004). According to this model, charged amino acid residues located inside the fibril, can destabilize this structure, due to the electrostatic repulsion. In this work, we investigated the effect of this mutation on the prion propagation and properties of Sup35 aggregates.

## 2. Materials and Methods

### 2.1. Strains, Media, and Growth Condition

*Saccharomyces cerevisiae* strain 7A-D832 [*psi*^–^] and its isogenic [*PSI*^+^] derivative 10-7A-D832 (Bondarev et al., 2013) were used in this study unless otherwise specified. Both strains contain the *sup35::TRP1* knockout on the chromosome, compensated by plasmid(s) bearing the *SUP35* gene. For the experiments with protein transformation, the [*psi*^–^] [*pin*^–^] strain 2-OT56 (Matveenko et al., 2016) was used.

Yeast strain 12-D1682, used for the induction of new [*PSI*^+^] variants, was constructed as follows. Strain GT671 was transformed by pRSU2 plasmid, carrying the *URA3* marker. Transformant with the Ura^+^Leu^–^ phenotype was selected and designated as U-GT671. Yeast strain GT159 (Chernoff et al., 1999) was transformed by the pRSU1 plasmid (Volkov et al., 2002), carrying *LEU2* marker, and mated with the U-GT671 strain. Diploids were selected on SC-Ura-Leu media. Then random ascospore isolates were obtained, and *MATa* Ura^–^Leu^+^ segregant was selected and named 12-D1682 (Table 1). This strain was transformed with a pRS316CUP-NM-GFP plasmid for overproduction of Sup35NM-GFP. The prion induction was performed as described below and seven prion variants were isolated. Clones that lost pRS316CUP-NM-GFP were selected after several passages on YEPD.

**Table 1:** Strains of *S. cerevisiae* used in this study.

**Strain**	**Genotype**	**References**
7A-D832	*MAT*α *ade1-14(UGA) his7-1(UAA) leu2 lys2-739 trp1 ura3 sup35::TRP1* [pYCH-U2] [*psi^–^*] [*PIN*^+^]	Bondarev et al., 2013
10-7A-D832	*MAT*α *ade1-14(UGA) his7-1(UAA) leu2 lys2-739 trp1 ura3 sup35::TRP1* [pYCH-U2] [*PSI*^+^] [*PIN*^+^]	Bondarev et al., 2013
2-OT56	*MAT***a** *ade1-14(UGA) trp1-289(UAG) ura3-52 his3-Δ200 leu2-3,112* [*psi^–^*] [*pin^–^*]	Matveenko et al., 2016
GT159	*MAT***a** *ade1-14(UGA) trp1-289(UAG) his3 lys2 ura3-52 leu2- 3,112* [*psi^–^*] [*PIN^+^*]	Chernoff et al., 1999
GT671	*MAT*α *ade1-14(UGA) trp1-289(UAG) his3 lys2 ura3-52 leu2- 3,112 sup35::HIS3MX* [*CEN LEU2 SUP35*] [*psi^–^*] [*pin^–^*]	Gift from Y.O. Chernoff
U-GT671	*MAT*α *ade1-14(UGA) trp1-289(UAG) his3 lys2 ura3-52 leu2- 3,112 sup35::HIS3MX* [pRSU2] [*psi^–^*] [*pin^–^*]	This study
12-D1682	*MAT***a** *ade1-14(UGA) trp1-289(UAG) his3 lys2 ura3-52 leu2- 3,112 sup35::HIS3MX* [pRSU1] [*psi^–^*] [*PIN^+^*]	This study
74-D694	*MAT***a** *ade1-14(UGA) trp1-289(UAG) ura3-52 his3-Δ200 leu2- 3,112* [*psi^–^*] [*PIN^+^*]	Derkatch et al., 1997
P-74-D694	*MAT***a** *ade1-14(UGA) trp1-289(UAG) ura3-52 his3-Δ200 leu2- 3,112* [*PSI^+^*] [*PIN^+^*]	Drozdova et al., 2016

Yeast cultures were maintained on the YEPD (yeast extract/peptone/dextrose medium) or synthetic complete minimal medium (SC) (Kaiser et al., 1994). Solid media were prepared with the addition of agar (2%). SC media with 5'-FOA (1 mg/ml) was used for counter-selection of plasmids bearing *URA3* (Kaiser et al., 1994). Nonsense suppression in [*PSI*^+^] cells was detected by the ability to grow on the SC medium lacking adenine (SC-Ade) or by the colony color on 1/4 YEPD (Eaglestone et al., 2000). Yeast cells were grown at 30°C.

### 2.2. Plasmids

Plasmids bearing the new *sup35* mutation were constructed by site-directed mutagenesis. We amplified the vector using highly processive DNA polymerase (AccuPrime Pfx, Invitrogen) (the primer sequences are available upon request). The vectors pRSU1 (Volkov et al., 2002), pRSU2 (Volkov et al., 2002), pRS316CUP-NM-GFP (Serio et al., 1999), pRS315CUP-NM-GFP and pET-20b-SUP35NM-His_6_ (Allen et al., 2005) were used as templates. Next, the PCR mixture was treated with DpnI (Thermo Scientific) to remove the template DNA. Then, this solution was used for transformation of *E. coli* competent cells. All mutations were verified by sequencing. To construct the pRS315CUP-NM-GFP plasmid, we ligated the region with the *CUP1* promoter, Sup35NM and GFP from pRS316CUP-NM-GFP (Serio et al., 1999) into the polylinker site of the pRS315 plasmid (Sikorski and Hieter, 1989). The region of interest in pRS316CUP-NM-GFP and the polylinker site were digested by XhoI and SacI enzymes. Sticky-end ligation was performed with T4 DNA-ligase according to Thermo Scientific protocol. pRS315CG was obtained analogously from pRS316CG (Serio et al., 1999) and pRS315. pR16CUP-NM-yTagRFP-T plasmid was obtained by insertion of the XhoI-XhoI fragment from pCUP-NM-His_6_ (Kiktev et al., 2015) in place of the XhoI-SalI fragment of pR16CUP-SFP1C-yTagRFP-T which in turn resulted from the substitution of the PstI-PstI fragment in pR16CUP-SFP1-Cerulean (Matveenko et al., 2016) for the PstI-PstI fragment from pIM35 (Malcova et al., 2016). TagRFP-T is a TagRFP derivative containing one additional substitution (Shaner et al., 2008). All the plasmids are listed in Table 2.

**Table 2:** Plasmids used in this study.

**Plasmid**	**Description**	**References**
pRSU1	*LEU2, ampR, PSUP35, SUP35*	Volkov et al., 2002
pRSU1-sup35-M0	*LEU2, ampR, PSUP35, sup35-M0*	This study
pRSU2	*URA3, ampR, PSUP35, SUP35*	Volkov et al., 2002
pRSU2-sup35-M0	*URA3, ampR, PSUP35, SUP35*	This study
pRS316CUP-NM-GFP	*URA3, ampR, PCUP1, SUP35NM-GFP*	Serio et al., 1999
pRS316CUP-NM-M0-GFP	*URA3, ampR, PCUP1, SUP35NM-M0-GFP*	This study
pET-20b-SUP35NM-His_6_	*-, ampR, T7, SUP35-NM-His_6_*	Allen et al., 2005
pET-20b-SUP35NM-M0-His_6_	*-, ampR, T7, SUP35-NM-M0-His_6_*	This study
pRS315CUP-NM-GFP	*LEU2, ampR, PCUP1, SUP35NM-GFP*	This study
pRS315CUP-NM-M0-GFP	*LEU2, ampR, PCUP1, SUP35NM-M0-GFP*	This study
pRS315	*LEU2, ampR*	Sikorski and Hieter, 1989
pRS315CG	*LEU2, ampR, PCUP1, GFP*	This study
pR16CUP-NM-yTagRFP-T	*URA3, ampR, PCUP1, SUP35NM-yTagRFP-T*	This study
pIM35	*URA3, ampR, PMET25, yTagRFP-T*	Malcova et al., 2016

*For each plasmid, the following characteristics are indicated: yeast selective marker, (“-” — the absence of a yeast selective marker), bacterial selective marker, promoter of the inserted gene, gene of interest. All yeast plasmids in the table are centromeric*.

### 2.3. Genetic and Microbiological Procedures

Standard microbiological approaches were used for all manipulations with yeast and bacterial colonies (Sambrook and Fritsch, 1989). Yeast protein transformation was performed as described previously (Tanaka and Weissman, 2006). Direct plasmid shuffle (from wild-type to mutant allele) was performed as follows: the [*PSI*^+^] *sup35::TRP1* strain with the *SUP35* gene on a *URA3* plasmid was transformed with *LEU2* plasmids bearing the wild-type or mutant *SUP35* alleles. Transformants, selected on SC medium lacking uracil and leucine (SC-Ura-Leu), were tested for suppression of the *ade1-14* mutation to determine the presence of [*PSI*^+^]. These transformants were replica plated on media with 5′-FOA for counter-selection of the plasmid with *SUP35*, and then on the SC-Leu and SC-Ura media to prove the loss of the plasmid. The suppressor phenotype of the obtained strains was analyzed on SC-Ade or 1/4 YEPD. Reverse plasmid shuffle (from mutant allele to wild-type) was performed as follows: the strains after direct shuffle were transformed with plasmids bearing the wild-type allele. Transformants were selected on SC-Ura-Leu medium and then streaked out on YEPD media to allow spontaneous plasmid loss. Colonies were replica plated on SC-Leu or SC-Ura medium to identify clones which contain only wild-type *SUP35* allele. After selection, the cells were tested for suppression of the *ade1-14* mutation to determine the presence of [*PSI*^+^].

The [*PSI*^+^] prion loss and transmission were scored according to the previously described procedure (Afanasieva et al., 2011) with minor modifications. The [*PSI*^+^] strain (10-7A-D832) was transformed with a *LEU2* plasmid bearing the *SUP35* or *sup35-M0*. To estimate [*PSI*^+^] curing, caused by the presence of mutated *sup35* allele, three transformants for each allele were replica plated three times on a medium lacking uracil, then resuspended in water and plated on 1/4 YEPD medium to obtain single colonies and to reveal the nonsense suppressor phenotype. Then these clones were replica plated on media lacking uracil or leucine. The frequency of prion loss was estimated as a fraction of Ura^+^Leu^–^ [*PSI*^+^] colonies. To determine the efficiency of [*PSI*^+^] transmission from Sup35 to Sup35-M0, 50 transformants for each combination of *SUP35* and *sup35-M0* alleles were replica plated three times on medium lacking leucine containing uracil to enable the cells to lose plasmid containing wild-type *SUP35*. To estimate the efficiency of [*PSI*^+^] transmission, the fraction of Ade^+^ colonies was scored amongst Ura^–^Leu^+^ isolates.

### 2.4. [*PSI*^+^] Induction

Plasmids bearing *SUP35NM-GFP* or *GFP* under control of *CUP1* promoter were used for the prion induction. Strains [*psi*^–^][*PIN*^+^] with corresponding plasmids were grown in selective media at 30°C to logarithmic phase. For the induction of *CUP1* promoter, CuSO_4_ was added into the media to the final concentration of 100 μM for the 7A-D832 strain or 50 μM for 12-D1682. Before induction and after 24 h, the aliquots of cultures were plated on 1/4 YEPD to count the number of white clones and evaluate the frequency of their appearance. To compare the amounts of Sup35NM for different constructions, other aliquots were taken at the same time points. Cell lysates were obtained with alkaline lysis (Zhang et al., 2011) and subsequently analyzed with SDS-PAGE (Sambrook and Fritsch, 1989). The same cells were used for fluorescence microscopy.

### 2.5. Decoration of Sup35 Aggregates *in vivo*

Two combinations of isogenic strains were used in this experiment: P-74-D694 and 74-D694, or 10-7A-D832 and 7A-D832. The first pair (P-74-D694 and 74-D694) was transformed with plasmids for production of Sup35NM fused with different fluorescent proteins (Sup35NM-TagRFP-T and Sup35NM-GFP with substitutions) and corresponding control constructs (TagRFP-T and GFP). For the TagRFP-T production, cells with plasmid pIM35 were grown overnight in the liquid media lacking methionine. For overproduction of the other constructs with fluorescent proteins, CuSO_4_ was added to a final concentration of 50 μM. The second pair of strains (10-7A-D832 and 7A-D832) was transformed with plasmids for production of Sup35NM-GFP, Sup35NM-M0-GFP or GFP. Overproduction of these proteins was induced by addition of CuSO_4_ to a final concentration of 100 μM. In all cases, the induction time was 4 h.

### 2.6. Propagon Counts

The transformants of 10-7A-D832 with pRS316CUP-NM-GFP and pRS316CUP-NM-M0-GFP were used for the propagon counts. The cells were grown in liquid SC-Ura medium with additional adenine to the early logarithmic phase (OD_600_ = 0.2). Then CuSO_4_ was added to a final concentration of 25 μM. Cells were plated on YEPD supplemented with 3 mM GuHCl to obtain single colonies before the addition of CuSO_4_ and after one cell culture division (estimated by OD_600_). The number of propagons in cells was determined using a previously described colony-based method (Cox et al., 2003).

### 2.7. Fluorescence Microscopy

Cells were gently pelleted (2000-3000 rpm) and resuspended in 50% glycerol. Fluorescence was analyzed using a Zeiss AxioScope.A1 wide-field fluorescence microscope. Images were taken with a QIClick-F-CLR-12 (QImaging) camera using QCAPTURE PRO 7 software.

### 2.8. Protein Analysis

The amount of Sup35 in different strains was quantified using Western Blotting with rabbit polyclonal anti-Sup35 antibodies (Chabelskaya et al., 2004). Monoclonal anti-tubulin antibodies (T6074, Sigma) were used for tubulin detection. Densitometry measurements were performed in ImageJ software (Schneider et al., 2012). SDS-PAGE with additional boiling (Kushnirov et al., 2006) was performed to detect Sup35NM-GFP and Sup35 in the aggregated and soluble fractions. For the analysis of Sup35 amyloid aggregates, SDD-AGE was used (Kryndushkin et al., 2003).

### 2.9. Protein Purification From *Escherichia coli* and Fibril Preparation

For Sup35NM purification, pET-20b-SUP35NM-His_6_ (Allen et al., 2005) plasmid or its derivative for Sup35NM-M0 overproduction were used. For protein purification, *E. coli* strain BL21(DE3) was used (Studier and Moffattf, 1986). Overproduction of recombinant proteins was carried out in 2TYa media with 1 mM IPTG. Cultures were grown at 37°C for 6 h. Proteins were purified in denaturing conditions (in the presence of 8 M urea) according to previously published protocols (Glover et al., 1997; Serio et al., 1999). The purification was performed with a two-step procedure with Ni-NTA agarose (Invitrogen) and Q-sepharose (GE Healthcare) columns. Proteins were concentrated with a centrifuge concentrator with molecular weight cutoff of 30 kDa (Millipore).

The obtained Sup35NM proteins were diluted at least 100-fold into fibril assembly buffer (5 mM potassium phosphate pH 7.5, 150 mM NaCl) to a final protein concentration of 0.5 mg/ml. In these conditions, Sup35NM spontaneously forms aggregates. Samples were incubated at 26°C with slow overhead rotation (rotator Bio RS-24, Biosan). To monitor amyloid fibril formation, aliquots were removed every 12 h up to 24 h of incubation. The rate of aggregated protein was estimated by SDS-PAGE with boiled and unboiled samples.

### 2.10. TEM and AFM

For fibrils visualization, Jeol JEM-2100 transmission electron microscope and Bruker Nanoscope V atomic force microscope were used. The negative staining with a 1% aqueous solution of uranyl acetate was used for TEM measurements. Samples were prepared by applying 5 μl of the Sup35NM fibril solution with concentration 0.5 mg/ml on a substrate, followed by washing with distilled water and drying. The fibrils were immobilized on freshly-cleaved mica surface for AFM analysis and formvar coated copper grids for TEM measurements (Sokolov et al., 2018).

### 2.11. Statistical Analysis

To compare the protein amounts the Mann-Whitney *U*-test was used (Mann and Whitney, 1947). The Fisher's exact test (Fisher, 1935) was used to compare the proportion of cells with a particular phenotype. All statistical tests were performed in R (R Core Team, 2018).

## 3. Results

### 3.1. Design of a New *sup35* Mutation

**Figure 1:**
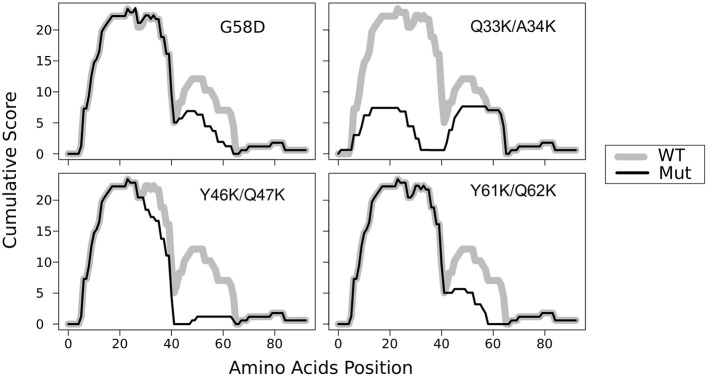
Substitutions Q33K/A34K within N-domain of Sup35 decrease the amyloidogenic potential of the protein. The ArchCandy program (Ahmed et al., 2015) was used to predict amyloidogenic properties. Cumulative scores (sum of β-arch scores counted for each amino acid residue) are presented on the plot. WT — wild-type protein; G58D, Q33K/A34K, Y46K/Q47K, and Q61K/Q62K substitutions that correspond to mutations *PNM2, sup35-M0, -M1*, and *-M2*, respectively.

In the previous work, we constructed five mutant *sup35* alleles, each of them leading to substitutions of two consecutive polar residues to charged ones (lysines) in the middle of one of the oligopeptide repeats (OR1 - OR5). Such mutations are incompatible with Sup35p aggregates with superpleated β-structure spanning the ORs with the respective mutations. These mutations were named *sup35^KK^*, and each was designated according to the number of ORs (from *sup35-M1* to *sup35-M5*) (Bondarev et al., 2013). We introduced mutations in all previously known ORs of Sup35 (Kushnirov et al., 1988). However, using T-REKS program, we identified additional repeat upstream of the known ORs (28–40 aa) (Bondarev et al., 2013). To complete the set of *sup35^KK^* alleles, we substituted two residues in the middle of newly identified OR0 (Q33K/A34K) to lysines and designated this mutation *sup35-M0*. The potential effect of this mutation on the Sup35 aggregation was evaluated with ArchCandy program (Ahmed et al., 2015). Previously it was shown that this tool accurately predicts the impact of amino acid substitutions on aggregation properties of a protein (Ahmed et al., 2015; Bondarev et al., 2015; Roche et al., 2017). According to the analysis, *sup35-M0* mutation could significantly decrease the amyloidogenic potential of Sup35 and have the highest effect on this parameter compared to known *PNM2* mutation (Doel et al., 1994) and other *sup35^KK^*, which were shown to eliminate the [*PSI*^+^] prion (Figure 1) (Bondarev et al., 2013).

### 3.2. The *sup35-M0* Mutation Efficiently Eliminates the [*PSI*^+^] Prion

**Figure 2:**
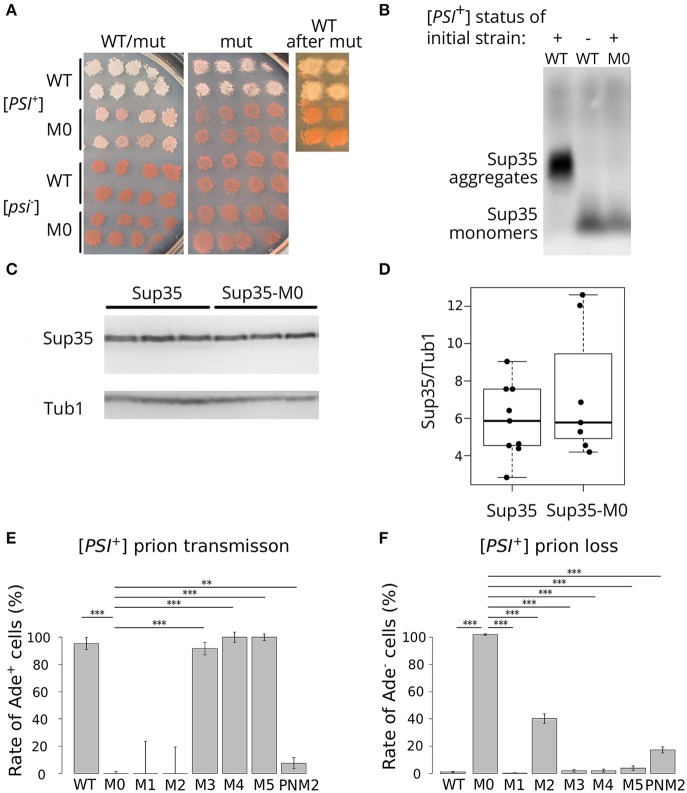
*sup35-M0* efficiently and irreversibly eliminates [*PSI*^+^] prion. **(A)** The phenotype of strains with different combinations of *SUP35* and *sup35-M0* alleles in [*PSI*^+^] and [*psi*^–^] strains on 1/4 YEPD is shown (images were taken after 4 days of incubation). Transformants bearing two plasmids with two wild-type alleles or combination of *sup35-M0* and *SUP35* are presented on the panel “WT/mut” (at least 16 transformants were analyzed). The phenotype of cells after the plasmid loss is shown on panel “mut”. Finally, *sup35-M0* (or *SUP35* as a control) were replaced with *SUP35* by the reverse plasmid shuffling, phenotype of obtained strains is presented on panel “WT after mut.” **(B)** The *sup35-M0* allele leads to the elimination of Sup35 aggregates according to SDD-AGE results. Antibodies against Sup35 were used for Western Blotting. **(C)** Result of Western Blot hybridization after SDS-PAGE analysis of protein lysates from the [*psi*^–^] strain with mutant or wild-type allele of *SUP35* with anti-Sup35 and anti-Tub1 antibodies. **(D)** The densitometry analysis of Sup35 protein level (ten replicates) revealed no difference in Sup35 protein level in strains with *sup35-M0* compared to *SUP35*. **(E)** [*PSI*^+^] transmission from the wild-type to the indicated *sup35* allele. Fraction of cells that retained the prion after loss of the wild-type allele is shown on graph. **(F)** [*PSI*^+^] loss induced by transient expression of the *sup35^KK^* alleles and *PNM2* mutation. Fraction of cells that have lost the prion after the loss of *sup35^KK^* allele is shown. ^**^p-value < 0.01 and ^***^p-value < 0.001 according to Fisher's exact test.

To analyze the effect of the mutation, we used previously described isogenic [*PSI*^+^] and [*psi*^–^] strains with *SUP35* deletion compensated by a copy of this gene on a *URA3* plasmid (Bondarev et al., 2013). In this system, we can change the alleles of *SUP35* by plasmid shuffling. The presence of the nonsense mutation *ade1-14* in these strains allows monitoring the prion propagation by the cell phenotype. As [*PSI*^+^] strains are able to suppress *ade1-14* mutation, we test for the prion loss by detecting the decrease in growth on medium lacking adenine accompanied by increased accumulation of the red pigment on 1/4 YEPD. To check the effect of *sup35-M0*, we transformed isogenic [*PSI*^+^] and [*psi*^–^] strains with a plasmid bearing *sup35-M0* or *SUP35* (control). All independent [*PSI*^+^] transformants bearing the mutant allele demonstrated a significant decrease in nonsense suppression phenotype on 1/4 YEPD (Figure 2A) or SC media without adenine (data not shown). The complete elimination of prion phenotype was observed after loss of the wild-type *SUP35* allele (Figure 2A). Yeast cells did not restore nonsense suppressor phenotype after replacement of a mutant allele by the wild-type (Figure 2A) suggesting that *sup35-M0* mutation leads to the [*PSI*^+^] prion loss.

Elimination of the prion should be accompanied by the elimination of Sup35 aggregates from the cells. We checked for the disappearance of aggregates in the transformants, which lost the wild-type *SUP35*, with SDD-AGE (Kryndushkin et al., 2003) and did not find Sup35 aggregates in cells bearing only *sup35-M0* (Figure 2B). This fact confirmed our assumption that the mutation eliminates the prion. We also compared the relative amount of the Sup35 protein for strains with the wild-type and the mutant *SUP35* allele and found no difference (Figures 2C,D), suggesting that the prion loss was not caused by a decreased amount of Sup35.

To compare the effects of the new mutation on the prion replication, we estimated the prion loss and transmission in the presence of *sup35-M0* allele according to special protocols for each parameter [(Afanasieva et al., 2011), see Materials and Methods section for details]. We did not observe cases of prion transmission to the *sup35-M0* allele (Figure 2E). At the same time, the rate of [*PSI*^+^] loss was 97.12 ± 0.55% that significantly exceeds the same parameter for other *sup35^KK^* mutations with a maximum value of ~40% in case of substitutions within the second OR (Figure 2F) (Bondarev et al., 2013). Based on this data we concluded that new mutation very efficiently eliminates the [*PSI*^+^] prion.

### 3.3. The Sup35NM-M0 Protein Forms Infectious Amyloid Aggregates

**Figure 3:**
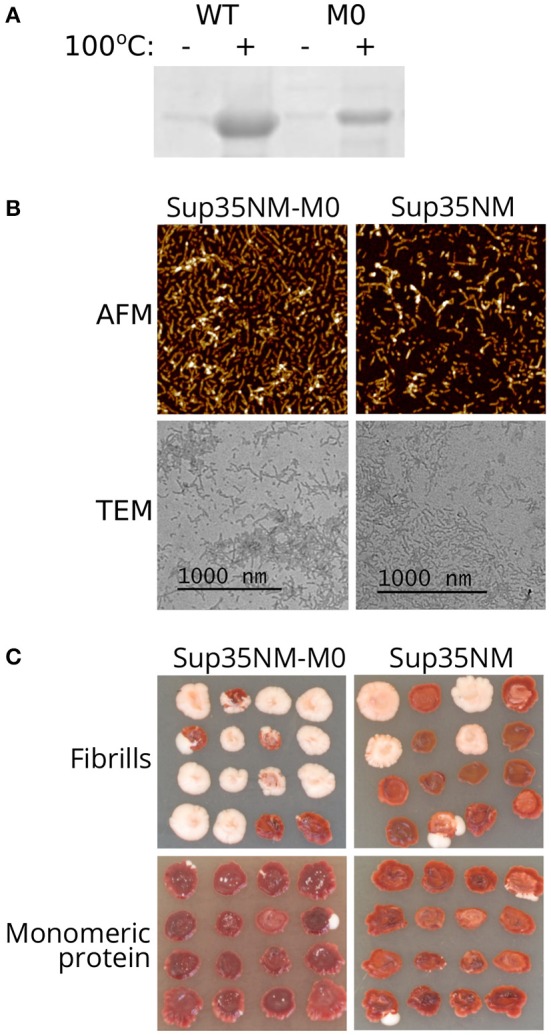
Sup35NM-M0 forms infectious amyloid aggregates. **(A)** Result of Coomassie staining of the gel after SDS-PAGE analysis of Sup35 fibrils formed *in vitro*. Sup35-M0 forms SDS-resistant aggregates similar to wild-type Sup35. **(B)** AFM and TEM images of fibrils formed by Sup35NM or Sup35NM-M0. **(C)** Phenotype of [*psi*^–^][*pin*^–^] strain (2-OT56) transformed with fibrillar or monomeric proteins on 1/4 YEPD (16 independent transformants are shown for each case, images were taken after 5 days of incubation). The appearance of [*PSI*^+^] phenotype (white color) after protein transformation suggested infectious properties of the aggregates.

The highly efficient loss of the prion caused by *sup35-M0* suggested that Sup35 with amino acid changes Q33K/A34K might be unable to form aggregates and induce [*PSI*^+^] formation. To check this hypothesis, we constructed plasmids for purification of the Sup35NM-M0 protein from *E. coli* cells. We chose only the N-terminal part of the protein because it is sufficient for aggregation (Glover et al., 1997). Wild-type Sup35NM protein was used as a control. After 24 h of incubation in nondenaturing conditions, both proteins formed SDS-resistant aggregates, detected by comparing the amount of the proteins in boiled and unboiled samples analyzed with SDS-PAGE (Figure 3A). Using atomic force microscopy (AFM) and transmission electron microscopy (TEM) we investigated the morphology of Sup35NM and Sup35-M0 aggregates and found no detectable difference between them (Figure 3B). To test infectious properties of the obtained fibrils, we used the protein transformation technique (Tanaka and Weissman, 2006) and demonstrated that aggregates of both proteins were infectious and led to [*PSI*^+^] appearance (Figure 3C). In all cases, the observed nonsense suppressor phenotype was prion-mediated because it was lost after cell growth on the media with GuHCl (data not shown), which is known to cause the loss of [*PSI*^+^] (Tuite et al., 1981). At the same time, cells transformed with monomeric protein did not acquire prion phenotype. Thus, charged residues within OR0 in Sup35 do not significantly change its ability to form infectious aggregates *in vitro*.

### 3.4. The *sup35-M0* Allele Can Induce and Propagate the [*PSI*^+^] Prion but With Low Efficiency

**Figure 4:**
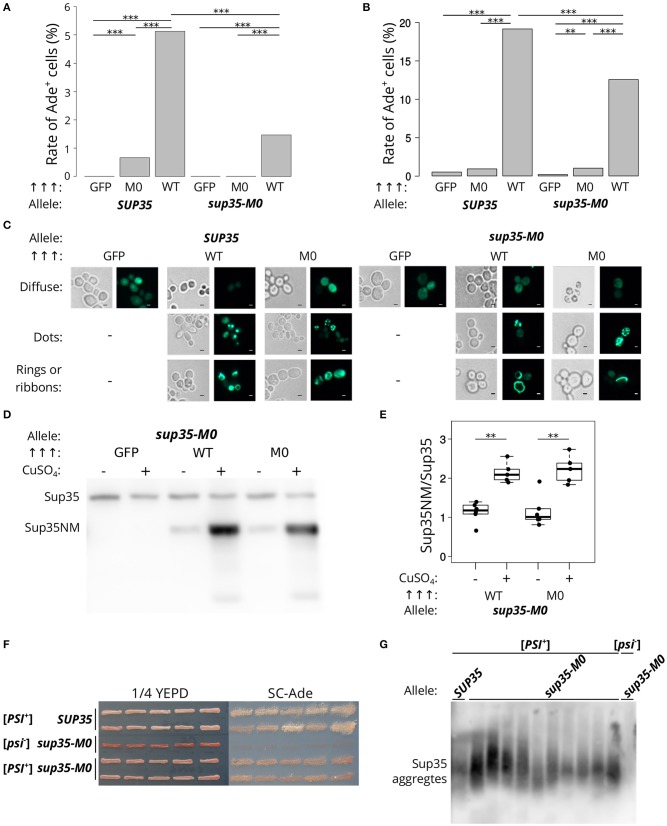
*sup35-M0* can induce and propagate [*PSI*^+^] prion but with low efficiency. **(A)** Frequencies of [*PSI*^+^] induction in the [*psi*^–^][*PIN*^+^] 7A-D832 **(A)** and 12-D1682 **(B)** cells upon overexpression of *SUP35NM-GFP* (WT) or *sup35NM-M0-GFP* (M0) in the presence of wild-type or mutant allele of *SUP35*. Overproduction of GFP hereafter was used as a negative control. All constructions were under control of *CUP1* promoter, CuSO_4_ was used for the 24 h induction. All experiments were repeated six times. Our results demonstrated that the mutation has a dramatically lower potential to induce [*PSI*^+^] prion than *SUP35* (^***^p-value < 0.001 according to Fisher's exact test). The “Allele” designates allele of full-length *SUP35* present in cells. **(C)** The cells tested on panel A were analyzed with the fluorescence microscopy (scale bar equals 5 μm). Various types of prion aggregates (dots, rings, and ribbons) were detected in the presence of both alleles (*SUP35* and *sup35-M0*). **(D)** Results of Western Blot hybridization after SDS-PAGE analysis of protein lysates of strains used for [*PSI*^+^] induction. **(E)** Densitometry analysis of the Western Blotting. The level of N-terminal domain of Sup35 fused to GFP was normalized to the full-length Sup35-M0 which is unchanged in cells with wild-type and mutant *sup35* allele according to the results presented on the Figure 2 (^**^p-value < 0.01 according to Mann-Whitney *U*-test). **(F)** The nonsense suppressor phenotype of several [*PSI*^+^] variants induced in presence of *SUP35* or *sup35-M0* in the 12-D1682 strain. Ten independent isolates are shown for each case. Cells were grown for 4 days on 1/4 YEPD and 5 days on SC-Ade. **(G)** The results of SDD-AGE analysis of protein lysates of typical [*PSI*^+^] variants induced in the presence of *sup35-M0*, antibodies against Sup35 were used for Western Blotting.

Next, we analyzed the effect of *sup35-M0* allele on the prion induction *in vivo*. The presence of another prion [*PIN*^+^] is required for [*PSI*^+^] *de novo* formation in yeast cells For this experiment [*psi*^–^][*PIN*^+^] strains (derivatives of 7A-D832 or 12-D1682 bearing *SUP35* or *sup35-M0*) were transformed with plasmids for Sup35NM-GFP (positive control), Sup35NM-M0-GFP or GFP (negative control) overproduction. The presence of *sup35-M0* in cells significantly decreased the frequency of [*PSI*^+^] formation in different yeast strains. Furthermore, the overproduction of Sup35NM-M0-GFP induced [*PSI*^+^] with very low efficiency (Figures 4A,B). This result allowed us to conclude that investigated substitutions significantly decrease the aggregation propensity of the protein, as was predicted by the ArchCandy program (Figure 1). We also compared patterns of Sup35NM-GFP and Sup35NM-M0-GFP fluorescence upon their overproduction. In all cases, we found that Sup35 aggregates formed during [*PSI*^+^] induction: rings, ribbons, or dots (Figure 4C), which are detected on the different stages of the prion life cycle (Tyedmers, 2012).

The low frequency of the prion induction upon *sup35NM-M0* overexpression may also be explained by the effect of the mutation on the protein stability. However, the levels of corresponding proteins upon their overproduction are the same (Figures 4D,E), which contradicts this hypothesis.

To check that the cells with nonsense suppressor phenotype and bearing *sup35-M0* were [*PSI*^+^] we isolated several corresponding clones of 12-D1682. The suppressor phenotype of these clones was preserved after several passages (Figure 4F) and in the absence of the plasmid, used for [*PSI*^+^] induction, but eliminated after the growth on GuHCl containing media (data not shown). We also found aggregates of Sup35 in all analyzed strains with prion variants (Figure 4G). These results prove the ability of *sup35-M0* to maintain the [*PSI*^+^] prion. Nevertheless, it should be mentioned that all [*PSI*^+^] variants formed in presence of *sup35-M0* had weak suppressor phenotype (Figure 4F).

### 3.5. The Sup35-M0 Protein Can Incorporate Into Fibrils of the Wild-Type Protein *in vivo*

**Figure 5:**
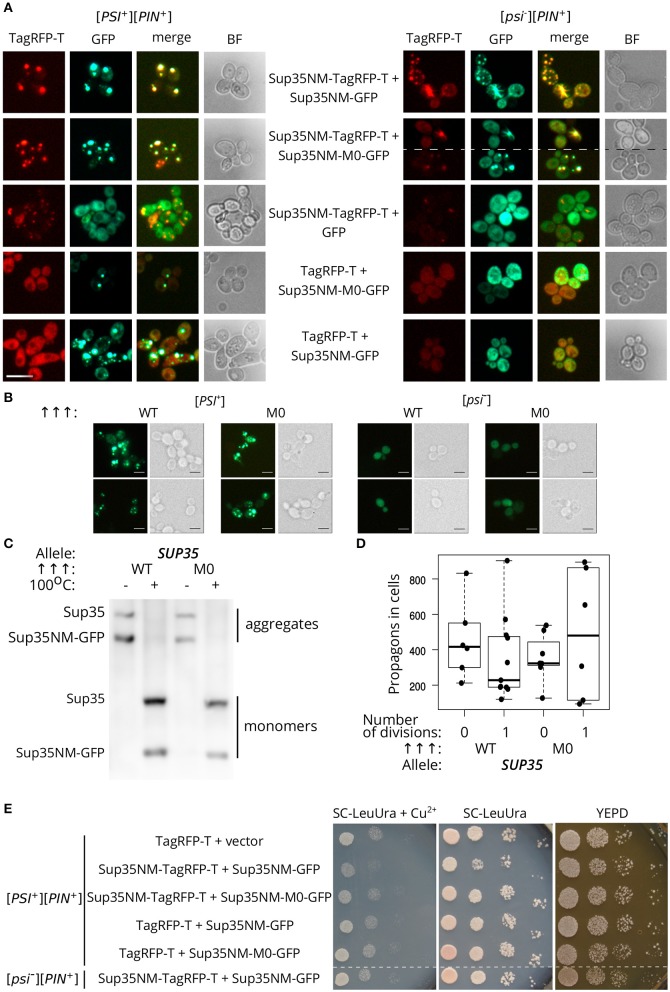
The Sup35-M0 protein can incorporate into fibrils of wild-type protein *in vivo*. **(A)** [*psi*^–^][*PIN*^+^] (74-D694) and [*PSI*^+^][*PIN*^+^] (P-74-D694) yeast strains were transformed with the plasmids for overproduction of Sup35NM-yTagRFP-T, in combination with either Sup35NM-GFP or Sup35NM-M0-GFP. We observed that the aggregates of Sup35NM and Sup35NM-M0 colocalize in [*PSI*^+^], as well as in [*psi*^–^] cells (scale bar equals 10 μm). **(B)** The transformants of [*psi*^–^][*PIN*^+^] (7A-D832) and [*PSI*^+^][*PIN*^+^] (10-7A-D832) with overproduced Sup35NM-GFP (WT), or Sup35NM-M0-GFP (M0) were analyzed with fluorescence microscopy (scale bar equals 5 μm). We detected foci of both proteins only in [*PSI*^+^], but not in [*psi*^–^], strain, which indicates inclusion of the proteins into existing aggregates. **(C)** The result of SDS-PAGE with boiled gel for strains from the panel B was shown. The “Allele” designates allele of full-length SUP35 present in cells. The Sup35NM-GFP (WT) and Sup35NM-M0-GFP (M0) proteins can incorporate into the existing prion aggregates upon transient overproduction in [*PSI*^+^] strain (both are detected in a fraction of aggregates). **(D)** Production of Sup35NM-M0-GFP does not affect the number of propagons. The cells from the panel B were used to calculate number of propagons before and after mild overproduction of Sup35NM-GFP or Sup35NM-M0-GFP; 25 μM CuSO_4_ was used for the induction. **(E)** Cells with overproduction of Sup35NM-TagRFP-T together with Sup35-NM-M0-GFP or Sup35NM-GFP were analyzed for prion toxicity. Cells were plated in 10-fold serial dilutions and grown for 2 days on SC-LeuUra + Cu^2+^ or YEPD and 4 days on SC-UraLeu. TagRFP-T production was used as a control; vector — pRS315.

We analyzed the ability of the protein with Q33K/A34K substitutions to incorporate into pre-existing Sup35 aggregates *in vivo*. Transient overproduction of Sup35NM fused with fluorescent protein leads to the decoration of existing Sup35 aggregates and formation of detectable fluorescent foci in cells (Osherovich et al., 2004). [*PSI*^+^][*PIN*^+^] and [*psi*^–^][*PIN*^+^] yeast strains (P-74-D694 and 74-D694, respectively) were transformed with the plasmids for overproduction of Sup35NM fused to a red fluorescent protein, TagRFP-T, in combination with either Sup35NM-GFP, or Sup35NM-M0-GFP, and analyzed with fluorescence microscopy. This experiment showed that the aggregates of Sup35NM and Sup35NM-M0 decorate [*PSI*^+^] aggregates (Figure 5A). One possible explanation of these results is that independently formed Sup35-M0 fibrils might co-localize with the wild-type fibrils, however, colocalization of the fibrils forming *de novo* in [*psi*^–^] strains seems less likely than co-aggregation of the wild-type and mutant proteins. Then we rechecked the ability of Sup35-M0 to embed into aggregates of the prion variant used in experiments with plasmid shuffle. The isogenic [*PSI*^+^][*PIN*^+^] and [*psi*^–^][*PIN*^+^] strains (10-7A-D832 and 7A-D832, respectively) were analyzed for the aggregate formation of Sup35NM-GFP or Sup35NM-M0-GFP. We detected fluorescent foci for both proteins only in [*PSI*^+^] strain (Figure 5B), which suggested the inclusion of the proteins into pre-existing aggregates, rather than *de novo* aggregation of the overproduced proteins. Then the incorporation of Sup35NM-M0 into amyloid aggregates was analyzed with SDS-PAGE with modifications, which allowed to evaluate the distribution of the protein with substitutions between fractions of detergent-resistant aggregates and monomers (Figure 5C). The results clearly demonstrated that Sup35NM-M0 was converted into amyloid-like conformation in the investigated [*PSI*^+^] strain. Taken together these data demonstrated the ability of the Sup35-M0 to incorporate into various Sup35 aggregates *in vivo*.

Incorporation of Sup35-M0 into the existing prion aggregates may have different effects. Previously we proposed that the analogous mutation in the second OR (*sup35-M2*) leads to formation of non-heritable fold and as a result to the prion loss (Bondarev et al., 2013). The main mechanism responsible for the prion transmission is a fragmentation of the prion aggregates by chaperones (Liebman and Chernoff, 2012). An impairment of this process should lead to the decrease in number of prion “seeds,” called propagons, and affects the transmission of the prion upon cell division. We analyzed the effect of Sup35NM-M0-GFP production on the propagon number after one generation and found no significant differences compared to Sup35NM-GFP (Figure 5D). Thus, we considered that *sup35-M0* has negligible effect on the aggregate fragmentation.

Finally, using phenotypic assay, we investigated the influence of the Sup35NM-M0 incorporation into wild-type Sup35 aggregates on the [*PSI*^+^] prion properties. One of the effects of increased Sup35 aggregation in [*PSI*^+^] cells is a reduction in cell viability as overproduction of Sup35NM in [*PSI*^+^] strains may lead to increased prion-dependent lethality (Derkatch, 1998; Vishveshwara et al., 2009). We checked whether Sup35NM-M0 retained the toxic properties of the wild-type protein. In contrast to Sup35NM-GFP, overexpression of Sup35NM-M0-GFP did not lead to a decrease in cell viability (Figure 5E). Overall, our data imply that the mutant Sup35 is able to co-aggregate with the wild-type protein, but their coaggregation may destabilize prion propagation of the native protein.

### 3.6. The Effect of *sup35-M0* Mutation Is Variant-Unspecific

**Figure 6:**
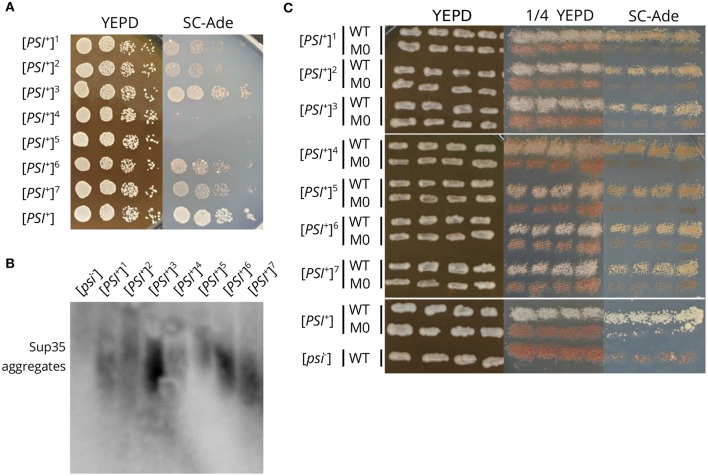
The *sup35-M0* mutation destabilizes different [*PSI*^+^] variants. **(A)** The suppressor phenotype of the obtained [*PSI*^+^] variants (designated by numbers 1-7). [*PSI*^+^] designates the 10-7A-D832 strain. Cells were plated in 10-fold serial dilutions and grown for 3 days on YEPD and 5 days on SC-Ade. **(B)** The comparison of Sup35 aggregate size in strains from the panel A. [*psi*^–^] is the 12-D1682 strain. **(C)** The suppressor phenotype of the same strains after the replacement of *SUP35* by *sup35-M0*. Cells were grown for 3 days on YEPD or 1/4 YEPD and 7 days on SC-Ade.

The effect of *sup35* mutations on [*PSI*^+^] usually depends on the prion variant (Derkatch et al., 1999; King, 2001). We checked whether the effects of *sup35-M0* are variant-specific. We obtained seven new [*PSI*^+^] prion variants in the strain with a single copy of *SUP35* on the plasmid (see Materials and Methods section for details). These strains had different strengths of the nonsense suppressor phenotype on medium lacking adenine, and differed in size of Sup35 aggregates (Figures 6A,B). The replacement of *SUP35* by *sup35-M0* in these strains led to the loss of nonsense suppressor phenotype (Figure 6C) and Sup35 aggregates (verified for two investigated strains with SDD-AGE, data not shown). This allowed us to conclude that the *sup35-M0* allele eliminates [*PSI*^+^] independently of the prion variant.

## 4. Discussion

The N-terminal domain of Sup35 is traditionally subdivided onto QN-rich (1–39 aa) and oligopeptide repeats regions (40–112 aa) (Kushnirov et al., 1988). The minimal region essential for [*PSI*^+^] propagation was assumed to comprise the first 57 residues, i.e., all QN region and two first ORs (Osherovich et al., 2004; Shkundina et al., 2006). In this work, we described the effects of substitutions (Q33K/A34K) within previously uncharacterized oligopeptide repeat of Sup35 on the [*PSI*^+^] prion propagation. We designated this new mutation as *sup35-M0* and showed that it is able to eliminate [*PSI*^+^]. This fact is in good agreement with position of the mutation, as well as the fact, that the majority of known PNM mutations is located within the region which was shown to be important for [*PSI*^+^] maintenance (DePace et al., 1998; King, 2001).

The *sup35^KK^* mutations, as it was shown previously, may have different effects on [*PSI*^+^] propagation (Bondarev et al., 2013). Taken together with current results, we can conclude that proteins with corresponding substitutions within OR0–OR2 eliminate the prion, but can incorporate into pre-existing prion aggregates, induce [*PSI*^+^] appearance and form amyloid aggregates *in vitro* (Bondarev et al., 2013 and *paper in prep*). Despite these common features only *sup35-M2* and the *sup35-M0* eliminate the prion even in presence of the wild-type allele (Figure 2F). This allows us to speculate that the role of first OR in the prion propagation is different from OR0 or OR2.

The hallmark of *sup35-M0* is the higher efficiency of [*PSI*^+^] elimination (97.12 ± 0.55%, Figure 2F) compared to the other previously characterized PNM mutations. For example, the prion loss in the presence of *PNM2* or *sup35-M2* mutations reachs 20 and 40%, respectively (Bondarev et al., 2013). The shuffle of *SUP35* from *S. cerevisiae* to the homologs from other yeast species (*S. paradoxus, S. bayanus, S. mikatae, S. kudriavzevii*) may also lead to the [*PSI*^+^] elimination but with lower efficiency than *sup35-M0* (Afanasieva et al., 2011). Furthermore, the prion elimination by the *sup35-M0* mutation can destabilize different [*PSI*^+^] variants (Figure 6C), while the effects of all previously described PNM mutations were variant-specific (Derkatch et al., 1999; King, 2001). Another specific feature of *sup35-M0* is a very low ability to induce and propagate the prion. Our results suggest that this mutation can maintain only limited number of weak prion variants (Figure 4F).

The strong effect of *sup35-M0* was not linked to the stability of the protein as the relative amounts of wild-type and mutant proteins did not differ (Figures 2C,D). Also, the elimination of the prion in the presence of *sup35-M0* could not be explained by the complete inability of the protein to propagate [*PSI*^+^]. Sup35NM-M0 can form infectious aggregates *in vitro* (Figure 3C), overproduction of Sup35NM-M0-GFP leads to the prion induction *in vivo*, and, finally, *sup35-M0* can maintain the prion, though, with low efficiency (Figures 4A,B). The low prion induction rate upon overproduction of Sup35NM-M0-GFP and in the presence of *sup35-M0* may be explained by the reduced ability of the soluble protein to aggregate with itself, as was demonstrated for Sup35-M1 and Sup35-M2 (Khan et al., 2018).

It is noteworthy that our experimental results once again illustrate the accuracy of ArchCandy prediction (Ahmed et al., 2015; Bondarev et al., 2015; Roche et al., 2017). We found that *sup35-M0* significantly decreases the frequency of the prion induction *de novo* and has lower prionogenic potential *in vivo* (Figure 4A). These data are in a good agreement with the bioinformatics predictions of ArchCandy, according to which lysines in 33–34 aa positions significantly decrease the amyloidogenic potential of Sup35 protein (Figure 1).

Prion loss caused by a certain *SUP35* allele may occur due to different mechanisms. In case of the interspecies barrier, three mechanisms were proposed: the inability of the heterologous protein to incorporate into prion aggregates, the block of aggregation by the protein and formation of the non-heritable fold of aggregates (Afanasieva et al., 2011). Detailed investigation of [*PSI*^+^] elimination caused by *PNM2* revealed two potential processes that may explain nonheritable properties of aggregates: increased fragmentation leading to solubilization of aggregates and impairment of prion transmission to the daughter cell (DiSalvo et al., 2011; Verges et al., 2011; Pei et al., 2017). We found that Sup35NM-M0 can incorporate into pre-existing Sup35 aggregates *in vivo* (Figures 5B,C), but this does not affect the number of propagons (Figure 5D) and thus the fragmentation of aggregates. It seems that both mechanisms could not explain the effect of *sup35-M0*. We suppose that the prion loss caused by *sup35-M0* is rather linked with the decreased aggregation propensity of the protein, followed by solubilization of aggregates by cellular chaperones. This hypothesis is in good agreement with very low [*PSI*^+^] induction rate in the presence of *sup35-M0* (Figure 4A) and prediction of the ArchCandy (Figure 1). However, this disagrees with the high efficiency of co-aggregation of Sup35NM-M0 with Sup35 (Figure 5), but we suggest that differences in aggregation rate in this experiment may be hidden due to the overproduction of the protein.

In summary, here we described a new mutation in *SUP35*, which can efficiently eliminate [*PSI*^+^] factor in a variant-independent manner. The *sup35-M0* possess very low amyloidogenic potential and can protect cells from the spontaneous appearance of the prion. We suggest that the investigated mutation may be widely used for fast and non-specific elimination of the [*PSI*^+^] prion or for design of yeast strains which almost never undergo transition to the [*PSI*^+^] state. Moreover, our discovery may serve as a proof of concept for the design of a prion-eliminating mutations using specific bioinformatic tools. In mammals at least two analogous mutations, eliminating the PrP prion in presence of the wild type allele, were described. Both of them lead to substitutions of polar residue to the charged one (Q167R or Q218K) Thus, our study supports the design of analogous mutations that could block propagation of mammalian prion and amyloid proteins and thus may be useful for amyloidosis therapy.

## Data Availability Statement

The datasets generated for this study are available on request to the corresponding author.

## Author Contributions

LD, AM, PS, and SB designed the experiments. LD, VR, AM, MB, OP, DL, PS, and SB performed the experiments. LD, VR, AM, PS, and SB prepared figures. LD, AM, and SB wrote original draft. LD, AM, MB, DL, PS, NK, AK, GZ, and SB performed review and editing the manuscript.

### Conflict of Interest

The authors declare that the research was conducted in the absence of any commercial or financial relationships that could be construed as a potential conflict of interest.
